# Clinical Profile and Hematologic Parameters of Children Under Five Years Presenting With Wheeze

**DOI:** 10.7759/cureus.106066

**Published:** 2026-03-29

**Authors:** Saroj K Tripathy, Medhagopal R. G., Sarthak Das, Archana Malik

**Affiliations:** 1 Pediatrics, All India Institute of Medical Sciences, Deoghar, Deoghar, IND; 2 Pulmonary Medicine, All India Institute of Medical Sciences, Deoghar, Deoghar, IND

**Keywords:** asthma, hematologic parameters, preschool children, under-five children, wheeze

## Abstract

Background

Asthma diagnosis in children under five remains a challenge because of frequent viral wheeze and the inability to perform spirometry. Asthma predictive scores, including the latest Global Initiative for Asthma (GINA) guidelines in children under five, are based on risk factors and clinical features. Hematologic parameters in this age group with asthma have not been explored, which might aid in the diagnosis. The objective of our study was to evaluate hematologic parameters and the clinical profile in children under five years with wheeze.

Methodology

A cross-sectional study of children with wheeze aged zero to five years was conducted at a tertiary care teaching institution from 1 January 2025 to 31 December 2025. Sociodemographic, clinical, and hematological parameters of children were assessed.

Results

Results were obtained from 120 children. The median age of children was 47 months (2-60 months), with a male-to-female ratio of 1.18:1. Cough, fever, fast breathing, and exercise intolerance were observed in 82.5%, 32.5%, 30.8%, and 11.7% of children, respectively. A positive family history of allergy or asthma was noted in 28.3% cases. Comorbidities such as allergic rhinitis, atopic dermatitis, and urticaria were associated with 15 (12.5%), 12 (10.0%), and nine (7.5%) children, respectively. Hematologic abnormalities such as anemia were observed in 40% of children, while leucocytosis was present in 41.66%, making it the most common abnormality. Fever, urticaria, and anemia were significantly associated with a higher probability of asthma.

Conclusion

Cough was the most common presenting symptom, while anemia and leucocytosis were the most frequent hematological abnormalities. Fever, urticaria, and anemia were significantly associated with higher asthma probability categories.

## Introduction

Asthma is a chronic inflammatory disease arising from heterogeneous gene-environment interactions. It is characterized by variable airway obstruction and bronchial hyperresponsiveness [1]. It is one of the leading causes of pediatric hospital admission and chronic disease-related school absenteeism [2]. Asthma diagnosis in children under five remains a challenge because of frequent viral wheeze and the inability to perform spirometry. In addition, over two-thirds of recurrent wheezers outgrow the disease by the second decade of life [3]. The Global Initiative for Asthma (GINA) 2024 guidelines have developed a probability of asthma diagnosis in children under five years of age based on history and clinical findings [4]. Risk factors linked to asthma also include allergic rhinitis, urticaria, food allergy, atopic dermatitis, gastroesophageal reflux disease (GERD), obesity, and psychiatric illness [4,5]. Asthma is broadly divided into two major endotypes, i.e., eosinophilic and non-eosinophilic, based on peripheral blood eosinophilia, sputum eosinophil counts, or fractional exhaled nitric oxide (FeNO) values, which impact treatment response and disease severity in adults and adolescents [6]. Similarly, complete blood count-derived inflammatory markers are associated with the risk of mortality in adults with asthma [7].

The estimated prevalence of asthma in children in India is around 8% [8]. Asthma predictive scores, including the latest GINA guidelines for children under five, are based on risk factors and clinical features and do not account for biomarkers. Hematologic parameters in this age group with asthma have not been explored, which might aid in the diagnosis. The objective of our study was to assess the clinical profile, hematologic parameters, and the evaluation of these parameters with the probability of asthma in children under five.

## Materials and methods

Study design

A cross-sectional study was conducted in a tertiary care teaching institution from 1 January 2025 to 31 December 2025 after obtaining institutional ethical clearance.

Patient recruitment

All children aged zero to five years attending the outpatient, inpatient, or emergency department with wheezing on examination were included in this study after obtaining appropriate consent. This study excluded children already diagnosed with asthma who were on corticosteroid therapy or any other controller therapy. Purposive sampling was used to recruit subjects.

Sample size calculation

Based on previous studies, the prevalence of asthma in children is around 8% [8]. The sample size was estimated based on asthma prevalence from previous studies; however, the primary outcome of this study was asthma probability categorization. Assuming a 10% dropout rate and a 95% confidence interval, the minimal sample size calculated using OpenEpi software (Open Source Epidemiologic Statistics for Public Health, Atlanta, GA, USA) is 114. The formula, $n = \frac{\mathrm{DEFF} \cdot N \cdot p (1 - p)}{d^2 / Z_{1-\alpha/2}^2 \cdot (N - 1) + p (1 - p)}$, was used for sample size estimation.

Operational definition

In children with wheezing, asthma likelihood was categorized according to GINA 2024 probability-based criteria for children under five. Asthma probability was categorized based on clinical features into few have asthma, some have asthma, and most have asthma. Peripheral eosinophil count ≥ 150/µL was taken as a cutoff for differentiating eosinophilic from non-eosinophilic asthma endotypes. Malnutrition was diagnosed using the WHO growth charts. Age-standardized normal cut-off values for hemoglobin (Hb), leukocyte count, and platelet count were used to diagnose anemia, leucocytosis/leukopenia, and thrombocytosis/thrombocytopenia.

Data collection

Patients' age, sex, religion, caste, anthropometry, chief complaint, present history, past history, and family history of allergy or asthma were recorded. Clinical examination findings were registered. A diagnosis of asthma was made based on clinical features and classified according to the GINA 2024 guidelines. Hematologic investigation findings, including Hb, total leukocyte count (TLC), white blood cell (WBC) differential count, and platelet count, were recorded. Investigations were done as part of a routine evaluation in children under five with wheezing, along with clinical evaluation. Other investigations, such as chest X-rays, total IgE, and specific IgE, were performed on a case-by-case basis to confirm the diagnosis. An investigation report of the absolute eosinophil count (AEC) was noted. Comorbidities/risk factors like obesity, gastrointestinal reflux disease, allergic rhinitis, atopic dermatitis, urticaria, food allergy, anaphylaxis, anxiety, depression, and obstructive sleep apnea, if any, were recorded. All patients were treated as per the GINA 2014 guidelines. All the collected data was recorded in a predesigned case record form.

Statistical analysis

Data from individual case record forms were entered into a tabular format in MS Excel (Microsoft Corp., Redmond, WA, USA). Baseline characteristics of all patients were expressed in mean ± SD. The prevalence of each asthma endotype was expressed as percentages. Anemia, leucocytosis, differential neutrophil, eosinophil, and platelet count (thrombocytosis/thrombocytopenia) were expressed as percentages based on standard cut-offs. Clinical features among the three asthma probability groups were compared using the chi-square test. The mean or median, Hb, TLC, and platelets were calculated with standard deviation. A p-value < 0.05 was taken as significant. Statistical analysis was performed using IBM SPSS Statistics for Windows, Version 31 (Released 2025; IBM Corp., Armonk, New York, United States).

## Results

A total of 120 children were included in the analysis. The median age of children was 47 months, ranging from 2 to 60 months. Males constituted 54.2% of the participants, while 45.8% were females with a male-to-female ratio of 1.18:1. Most children had normal nutritional status (73.3%), whereas 24.2% were undernourished and 2.5% were obese. Cough was the predominant presenting symptom observed in 82.5% children. Other presenting symptoms, such as fever, fast breathing, and exercise intolerance, were observed in 32.5%, 30.8%, and 11.7% of children, respectively. Exclusive breastfeeding was observed in 102 (85%) children, and 115 children were born at term. A positive family history of allergy or asthma was noted in 28.3% cases. Children from poor socioeconomic backgrounds (below the poverty line, or BPL) constituted 48.3% of the total cases. Comorbidities such as allergic rhinitis, atopic dermatitis, and urticaria were associated with 15 (12.5%), 12 (10.0%), and nine (7.5%) children, respectively. Sociodemographic and clinical characteristics of children under five with wheeze are summarized in Table 1.

**Table 1:** Sociodemographic and Clinical Characteristics of Children Under Five Years With Wheeze BPL: below the poverty line

Characteristics	N (%)
Age in months (median (range))	47 (2-60)
Sex (M/F)	65 (54.2)/55 (45.8)
Nutritional status	
Normal	88 (73.3)
Undernutrition	29 (24.2)
Obesity	3 (2.5)
Fever	39 (32.5)
Cough	99 (82.5)
Fast breathing	37 (30.8)
Exercise intolerance	14 (11.7)
Term	115 (95)
Exclusive breastfeeding	102 (85)
Allergic rhinitis	15 (12.5)
Atopic dermatitis	12 (10.0)
Urticaria	9 (7.5)
Family history of allergy or asthma	34 (28.3)
Poor socioeconomic status (BPL)	58 (48.3)

The mean (SD) Hb level among under-five children with wheeze was 10.60 (1.46) g/dL. The median TLC was 10,000/mm^3^ with an interquartile range (IQR) of 8,325-13,000/mm^3^. The median platelet count was 2.99 lakhs/mm^3^ (IQR: 2.00-3.98), and the median AEC was 233/mm^3^ (IQR: 120-420). The mean or median, Hb, TLC, and platelet counts were within the normal range. Although mean hematologic values were within normal ranges, a substantial proportion of children met criteria for anemia (40%) and leucocytosis (41.66%). Thrombocytosis was found in 18.3% of the participants. Laboratory parameters and values are presented in Table 2.

**Table 2:** Hematologic Parameters in Children Under Five Years With Wheeze * mean ± SD; Hb: hemoglobin; TLC: total leucocyte count; AEC: absolute eosinophil count; IQR: interquartile range

Laboratory Parameters	Mean (SD)/Median (IQR)
Hb (gm/dL)	10.60 (1.46)*
TLC (per mm^3^)	10000 (8325-13000)
Platelet count (lakhs/mm^3^)	2.99 (2.00-3.98)
AEC (per mm^3^)	233 (120-420)
Hematologic Abnormalities	N (%)
Anemia	48 (40)
Leucocytosis	50 (41.66)
Thrombocytosis	22 (18.3)

Clinical characteristics and laboratory parameters were compared based on the probability of asthma subtypes. Nutritional status was normal in the majority of children with all subtypes. Nutritional status was not significantly associated with the probabilities of any asthma subtypes. Although female sex was more commonly associated with most asthma subtypes, it was not found to be significant (P = 0.571) when compared with those who have “few have asthma” subtypes, and “some have asthma" subtypes. Fever was significantly associated with the probability of asthma (χ^2^ = 6.610, p = 0.037). Among children with fever, 17.9% had the highest probability of asthma, compared to 11.1% among those without fever. Forty percent of children with urticaria belonged to most have asthma subtypes as compared to 10.9% without urticaria within this subgroup. Urticaria was also significantly associated with asthma probability (χ^2^ = 7.420, p = 0.024), suggesting that children with urticaria were more likely to belong to the higher asthma probability categories (Table 3).

**Table 3:** Comparison of Clinical and Laboratory Characteristics by Probability of Asthma in Children Under Five Years With Wheeze EBF: exclusive breastfeeding; EA: eosinophilic asthma * P-value <0.05 is significant.

Variables	Characteristics	Few Have Asthma (N=67), N (%)	Some Have Asthma (N=37), N (%)	Most Have Asthma (N=16), N (%)	Chi-Square Value	P-value*
Nutritional status	Normal	46 (52.3)	28 (31.8)	14 (15.9)	4.142	0.387
	Underweight	18 (62.1)	9 (31.0)	2 (6.9)		
	Obesity	3 (100)	0 (0)	0 (0)		
Sex	Male	36 (55.4)	22 (33.8)	7 (10.8)	1.122	0.571
	Female	31(56.4)	15 (27.3)	9 (16.4)		
Fever	Present	26 (66.7)	6 (15.4)	7 (17.9)	6.610	0.037
	Absent	41(50.6)	31 (38.3)	9 (11.1)		
Cough	Present	53 (53.5)	31 (31.3)	15 (15.2)	1.980	0.372
	Absent	14 (66.7)	6 (28.6)	1 (4.8)		
Fast breathing	Present	19 (51.4)	12 (32.4)	6 (16.2)	0.570	0.752
	Absent	48 (57.8)	25 (30.1)	10 (12.0)		
EBF	Yes	58 (56.9)	32 (31.4)	12 (11.8)	1.448	0.485
	No	9 (50.0)	5 (27.8)	4 (22.2)		
Family history of allergy/asthma	Yes	23 (67.6)	8 (23.5)	3 (8.8)	2.730	0.255
	No	44 (51.2)	29 (33.7)	13 (15.1)		
Urticaria	Yes	5(50.0)	1 (10.0)	4 (40.0)	7.420	0.024
	No	62 (56.4)	36 (32.7)	12 (10.9)		
Allergic rhinitis	Yes	8 (53.3)	5 (33.3)	2 (13.3)	0.054	0.973
	No	59 (56.2)	32 (30.5)	14 (13.3)		
Atopic dermatitis	Yes	5 (41.7)	4(33.3)	3 (25.0)	1.867	0.393
	No	62 (57.4)	33 (30.6)	13 (12.0)		
Anemia	Yes	33 (68.8)	9 (18.8)	6 (12.5)	6.221	0.045
	No	34 (47.2)	28 (38.9)	10 (13.9)		
Leucocytosis	Yes	29 (60.4)	10 (20.8)	9 (18.8)	4.656	0.097
	No	38 (52.8)	27 (37.5)	7 (9.7)		
Thrombocytosis	Yes	11 (47.8)	9 (39.1)	3 (13.0)	2.126	0.713
	No	56 (57.7)	28 (28.9)	13 (13.4)		
EA	Yes	41(50.0)	29 (35.4)	12 (14.6)	3.632	0.163
	No	26 (68.4)	8 (21.1)	4 (10.5)		

Among laboratory parameters, anemia demonstrated a statistically significant association with asthma probability (χ^2^ = 6.221, p = 0.045). Children with anemia showed a relatively higher distribution across higher asthma-probability groups than non-anemic children. Other variables, including nutritional status, sex, cough, fast breathing, exclusive breastfeeding, family history of allergy/asthma, allergic rhinitis, atopic dermatitis, leucocytosis, thrombocytosis, and eosinophilia, did not show statistically significant associations with asthma probability (p > 0.05) (Table 3).

## Discussion

Wheezing, a common respiratory presentation in early childhood, is a major cause of pediatric outpatient visits and hospitalization. The present study evaluated the sociodemographic profile, clinical characteristics, and hematological parameters among children under five years with wheeze and analyzed their associations with different asthma probability categories.

In our study, the median age of children with wheeze was 47 months. This finding indicates that wheezing episodes were more common during the preschool period. Wheezing is common in early childhood due to anatomical and functional immaturity of the respiratory tract. Studies have demonstrated that nearly one-third of children experience at least one wheezing episode during the first three years of life [9]. The Tucson Children’s Respiratory Study described distinct wheezing phenotypes in early childhood, including transient early wheezers, non-atopic wheezers, and persistent wheezers, who have a higher likelihood of developing asthma later in life [10]. Similar age distributions have been reported in several studies examining wheezing disorders among preschool children [11,12]. Most children in the present study had a normal nutritional status. There was no statistical significance of nutritional status with the probability of asthma in the present study. Malnutrition has been recognized as an important contributor to childhood respiratory morbidity, particularly in developing countries. Undernourished children are more susceptible to infections due to impaired immune responses, which may increase the frequency of respiratory illnesses and wheezing episodes [13]. On the other hand, obesity has emerged as an important risk factor for asthma in developed countries. Obesity-related asthma is thought to occur due to systemic inflammation, increased oxidative stress, and altered lung mechanics [14]. However, the very low percentage of obesity in the present study population may be a contributory factor for the lack of a significant change between obesity and asthma probability.

Cough was the most common presenting symptom in the present study, observed in 82.5% of children. Cough often occurs with wheezing and indicates airway inflammation and bronchial hyperresponsiveness. Cough has consistently been linked with wheezing in most children [15,16]. Coughing during crying or playing has been a subtle sign of asthma, particularly in young children [17]. Other symptoms noted in the study included fever (32.5%), rapid breathing (30.8%), and exercise intolerance (11.7%). Among these, fever was statistically significantly associated with the likelihood of asthma (p = 0.037). Respiratory infections are the leading triggers of wheezing episodes in young children. Viruses such as respiratory syncytial virus (RSV), parainfluenza virus, rhinovirus, and human metapneumovirus are key causes of wheezing illnesses [18]. Serious viral lower respiratory tract infections during infancy have been associated with an increased risk of recurrent wheezing and asthma later in childhood [19]. The link between fever and the likelihood of asthma observed in this study may therefore indicate that infection-related airway inflammation triggers wheezing episodes.

Exclusive breastfeeding was reported in 85% of children in the present study, and most were born at term. Breastfeeding is known to provide immunological protection against respiratory infections through antibodies, cytokines, and other bioactive factors in breast milk [20]. Several studies have suggested that exclusive breastfeeding during the first six months of life may reduce the incidence of early childhood respiratory infections and wheezing episodes [21]. However, in the present study, exclusive breastfeeding was not statistically significantly associated with the probability of asthma. This finding is consistent with some previous studies reporting mixed evidence regarding the protective effect of breastfeeding against the development of asthma later in childhood [22]. In addition, multiple factors play a role rather than being an isolated phenomenon in the causation of wheezing.

A positive family history of allergy or asthma was observed in 28.3% of children in this study. Genetic predisposition plays a significant role in the development of asthma. A family history of asthma is strongly associated with wheezing and asthma [23]. The Asthma Predictive Index (API) considers parental asthma as one of the major criteria for predicting persistent asthma in preschool wheezers [24]. However, our study did not find a statistically significant link between asthma probability and family history. This may be due to several factors, including the relatively small sample size and potential underreporting of family history by caregivers.

Allergic conditions, including allergic rhinitis, atopic dermatitis, and urticaria, were present in 12.5%, 10%, and 7.5% of children, respectively. These findings are consistent with the concept of the “atopic march,” which describes the progression of allergic diseases during childhood from eczema and food allergies to allergic rhinitis and asthma [25]. Children with urticaria were more likely to belong to the “most likely asthma” category compared to those without urticaria. Previous studies have similarly reported strong associations between allergic diseases and persistent wheezing in children [26]. Allergic conditions share common immunological pathways involving IgE-mediated hypersensitivity and eosinophilic airway inflammation, which contribute to asthma pathogenesis. Urticaria, as an important comorbidity in asthma, has also been reported in a previous study [27].

Although the mean value was within normal limits, anemia was observed in 40% of children. Anemia showed a statistically significant association with asthma probability (p = 0.045), with anemic children more frequently falling into the “most have asthma” probability categories. Iron-deficiency anemia may affect respiratory health through several mechanisms. Chronic hypoxia associated with anemia may also exacerbate respiratory symptoms and airway inflammation. Elevated leukocyte counts may reflect underlying infections or inflammatory processes commonly associated with wheezing illnesses in young children. However, leucocytosis was not statistically significantly associated with the probability of asthma. Similarly, thrombocytosis did not demonstrate a significant association with asthma probability. Platelets have been implicated in airway inflammation and asthma pathogenesis through interactions with leukocytes and endothelial cells [28]. However, the clinical significance of thrombocytosis in preschool wheezing remains unclear. The median AEC in this study was 233/mm^3^. Although eosinophilia is commonly associated with allergic asthma, it was not statistically significantly associated with the probability of asthma. This may reflect the predominance of infection-related wheezing in early childhood rather than allergic asthma. High peripheral blood eosinophilia across all subgroups could also be attributed to a higher risk of parasitic infection due to poor hygiene.

Predicting which preschool wheezers will develop persistent asthma remains a major challenge in pediatric practice. Several predictive models have been developed to identify high-risk children. The API is one of the most widely used tools and includes factors such as parental asthma, eczema, allergic rhinitis, and eosinophilia [23]. Children who meet the criteria of the API have a significantly increased risk of developing asthma during school age. Models incorporating demographic, clinical, and laboratory features have failed to correctly identify asthma in this population subgroup [29]. The 2024 GINA guidelines sought to classify wheezing in children under five into three categories to improve prediction and guide treatment [4]. However, no single model has achieved perfect predictive accuracy due to the complex, multifactorial nature of asthma development. Recent research has highlighted novel biomarkers and asthma endotypes in preschool wheeze, with implications for diagnosis and management [30]. The findings of our study highlight the importance of comprehensive evaluation in children presenting with wheeze. Identification of factors such as fever, allergic manifestations, and anemia may help clinicians identify children at increased risk of asthma. Early identification of high-risk children allows for timely interventions, including monitoring, environmental control measures, and appropriate pharmacological therapy.

Limitations

The present study has some limitations. First, the study's cross-sectional design failed to establish causal relationships between the identified factors and asthma development. Longitudinal follow-up studies are required to determine whether children with higher asthma probability eventually develop persistent asthma. Second, the study was conducted at a single tertiary care centre and had a relatively small sample size, which may not be generalizable. Third, environmental factors, such as exposure to tobacco smoke, indoor air pollution, and allergens, were not extensively evaluated.

## Conclusions

The present study provides valuable insights into the sociodemographic, clinical, and hematological characteristics of children under five presenting with wheeze. Cough was the most common presenting symptom, while anemia and leucocytosis were the most frequent hematological abnormalities. Fever, urticaria, and anemia were significantly associated with higher asthma probability categories. Future studies should use longitudinal designs to determine causal relationships among anemia, fever, urticaria, and asthma risk in children under five years old. The incorporation of objective biomarkers and diagnostic tools may improve the early identification and classification of asthma. Additionally, larger, multicenter, interventional studies are needed to evaluate the impact of correcting modifiable factors, such as anemia and infections.

These findings emphasize that wheezing disorders in early childhood are influenced by multiple factors, including infections, allergic predisposition, and nutritional status. Early identification of children at increased risk of asthma through clinical evaluation and simple laboratory investigations may facilitate timely intervention and improve long-term respiratory outcomes.
